# 

**Published:** 1845-09-01

**Authors:** 


☞ An enumeration of circulars from Medical Schools received since our last No. with brief notices, which we had prepared, and some editorial matter already in type, are necessarily deferred for want of space: also acknowledgments of some publications with which we have been favored. We have received, as additions to our exchanges, the Southern Med. and Surg. Journal, Augusta, Ga., and the British American Jour. of Med. and Physical Science; both valuable accessions to our list. Our readers will perceive that we endeavor to make the most of our space. In doing so we are obliged to presume somewhat on the well known complaisance of our Publisher. Compression into a given space of long primer and brevier, however, we find, has its limits which it is impossible to transcend.

